# Supplementary material for: Are carbon emissions trading and green financial instruments synergistic? -Comprehensive quantitative research based on content analysis
Source: PLoS One. 2024 Mar 7;19(3):e0298601. doi: 10.1371/journal.pone.0298601 (PMC10919601; doi:10.1371/journal.pone.0298601)
Supplement: S1 Data — (ZIP) [file pone.0298601.s001.zip › Data/地级市Z_F工作报告.zip!/╣π╢1⁄2/╜¡├┼╩╨/└┤╫╘╣1⁄2╓┌║┼í╛┬φ┐╦╩2╛▌═°í┐_2016.docx]

各位代表：

　　我代表市政府向大会作工作报告，请予审议，并请政协委员和列席人员提出意见。

　　“十二五”时期工作回顾

　　“十二五”时期，市政府在省委、省政府和市委的正确领导下，在市人大及其常委会的监督和市政协的支持下，主动适应新常态，落实稳增长、促改革、调结构、惠民生各项举措，地区生产总值突破2200亿元，经济社会保持了平稳较快发展，成为全国文明城市、全国小微企业创业创新基地示范城市、珠三角国家自主创新示范区的重要一员，全面完成了“十二五”规划和2015年目标任务。

　　一、明确使命，拓开新局

　　通过“十二五”尤其是2015年全市上下的共同努力，各级各部门以及广大干部群众统一了共识，凝聚了力量，为江门长远发展打下更坚实基础。

　　明确加快发展的新认识。全市上下着力抓好“种树、搭桥修路、抓大项目”三件大事，特别围绕省委、省政府赋予江门“珠西战略”策源地、主战场和增长极的新定位，把加快发展作为第一位的任务，紧盯实体经济与创新经济，抢抓发展机遇，整合发展资源，打造发展平台，2015年生产总值增长8.4%，高于全省平均水平。

　　明确“三门”[1]“三心”[2]的新使命。实施“五个主动对接”[3]，推动“高、大、中、小”四轮驱动[4]，开展“双引双创”[5]，引领江门再出发，打造开放之门、方便之门、辐射之门，建设珠西新的经济中心、创新中心和城市中心。

　　明确“东联西拓”的新重点。充分发挥我市区位优势，持续向东主动对接广佛都市圈、深港澳经济圈两大龙头，向西传递省战略西拓的能量，做好“传”“接”的“中卫”角色。

　　明确“东提西进”[6]的新格局。深化“三边”战略，按照东部一体、西部协同原则，以东西板块统筹制订发展政策，东部三区一市以“一核心两门户”[7]格局做强中心，西部以台开快速路为突破口推进台开同城，推动台开恩协同共进。

　　二、交通突破，产城共进

　　开展交通大会战，建成江顺大桥、江门大道北、广珠铁路、新台高速南延线、佛开高速九江大桥至三堡段扩建等一批基础设施，江门大道中、江门大道南及广中江、江罗、中开、高恩等高快速路，深茂铁路和高新区码头加快建设，珠西综合交通枢纽完成初步规划定位。出台实施珠西行动、创新驱动、农业强市、旅游强市等工作纲要及行动方案和“经九条”[8]等政策措施，推动制造、旅游、农业“三个大市”向“三个强市”转变。工业技改投资连续两年增长超过100%，增幅排在全省前列，建立4个省市共建战略性新兴产业基地，广东南车获全省唯一的动车组制造许可证，“江门制造”城际动车组交付使用；推进全域旅游，成为全省旅游综合改革示范市，全市旅游总收入达336亿元，连续四年增长超过20%，建立台开恩旅游联盟，全面启动步行径建设；新增农民专业合作社1315家、市级以上重点农业龙头企业30家，建设高标准基本农田112.5万亩，创建全国首批国家现代农业示范区。广东（江门）大广海湾经济区获批成为全省面积最大的省级经济区，江门产业转移工业园连续四年获省考评优秀等次，集中资源和力量建设“1+6”核心园区[9]，实现集聚发展、错位发展。“敲门招商”取得显著成效，引进投资额超亿元先进（装备）制造项目129个。

　　三、小微双创，创新引领

　　抢抓全国小微企业创业创新基地示范城市机遇，制定实施“雏鹰计划”[10]及“1+15”政策体系[11]，在全国首创建立小微企业和科技型小微企业名录库，成为全省唯一的小微企业创业创新综合改革试点市，中欧（江门）中小企业国际合作区、中国（江门）“侨梦苑”华侨华人创新产业聚集区、“青创汇”等“双引双创”平台落户。自主创新能力增强，“珠西智谷”“珠西创城”等创新载体加快建设。新增国家高新技术企业118家、国家级创新平台5家、省级工程技术研究中心76家、省市级院士工作站2家、新型研发机构13家，成为全国科技进步考核先进市和国家知识产权试点城市，成立市技术交易中心、科技金融综合服务中心、知识产权维权中心，江门中微子实验站启动建设。金融生态环境不断优化，贷款余额增长115%，成为广东省唯一的全国小微企业信用体系建设试验区、全国首个与澳门签订金融合作备忘录的地级市，新增境内外上市企业7家、新三板挂牌企业8家。

　　四、深化改革，主动作为

　　实施政府清单管理，全面公布实施市、县两级“1+3”清单[12]，在全省率先出台涉企行政事业性、经营服务性收费目录清单。深入推进商事制度改革，在全省地级市中首推“五证合一、一照一码”[13]，市场主体总数超25万户。全市共调整行政审批事项3264项，精简比例达43%。建设启用江门跨境电子商务快件分拣清关中心，成为全国首个跨境电子商务领域“单一窗口”。网上办事大厅实现省、市、县三级互联互通。国资整合成果进一步稳固，充分利用政府债务限额管理，有效保障了重大基础设施建设投融资开展。“大民政”扎实推进，新增社会组织2780个，成为省基本公共服务均等化综合改革试点。全面完成新一轮政府职能转变和机构改革，不断深化大部门制改革、综合行政执法改革、简政强镇事权改革，推进江门高新区、滨江新区、大广海湾经济区及有关区（市）的园区体制机制的调整完善，推动部分镇街整合工作。

　　五、改善民生，实事惠民

　　启动公园城市建设和国家森林城市创建工作，开展圭峰山“岭南文化名山”规划建设，新增森林公园32个、湿地公园4个，新建改建村居公园330个，建成城市绿道440公里，城市人均公园绿地面积达17.62平方米。全面完成省下达的“十二五”节能减排任务。环境质量总体保持稳定，全力推动潭江水环境综合整治、大气污染防治各项工作，城镇生活垃圾无害化处理率达100%。841条村庄开展整治工作。启动“33墟街”“南岸印迹”“学宫文心”规划保护。打响“中国侨都”城市品牌，以五邑银信为重要组成部分的《侨批档案》入选世界记忆遗产名录，中国侨乡文化研究中心落户，荣获“中国舞蹈之城”称号，打响江门健走马拉松等“四个一”[14]文体惠民品牌。

　　坚持创文惠民，每年办好十件民生实事，公共服务投入持续增长，各级财政民生支出五年累计571.9亿元。城乡居民收入累计增长75%。推动精准扶贫、精准脱贫，全面实现贫困村基本脱贫，全市1323个村（社区）全面建成公共服务站，全面完成省下达扶贫开发任务。全市城乡居民基本养老保险基础养老金标准、城乡低保标准、特困人员供养标准、孤儿供养水平、城乡医疗救助水平、低保残疾人生活津贴和重度残疾人护理补贴标准逐年提高。建立了城乡一体化的居民社会养老保险制度。超额完成省下达的保障性住房建设和华侨农场、农村危房改造任务。教育现代化先进市建设扎实推进，成为全省首批所有区（市）通过国家义务教育发展基本均衡县验收的地级市之一。建成一批国家、省和市卫生镇村，基层医疗卫生服务体系和食品药品安全治理体系不断完善。推动东部公交一体。获得“全国双拥模范城”五连冠。“平安江门”建设力度持续加大。各项生产安全事故持续下降，防御多次强台风强降雨实现无人员伤亡。外事、港澳、对台、民族、宗教、人防、打私、统计、审计、妇女儿童、新闻、出版、方志、气象、红十字会、应急等工作均有新的进展。

　　六、强化法治，政府提效

　　立足法治、透明、诚信、效率政府建设，营造良好政务环境。共办理人大代表建议635件，政协提案739件。被评为全国法治城市、法治县（市、区）创建活动先进单位，成为全省首批制定地方性法规的地级市之一。认真开展党的群众路线教育实践活动、“三严三实”[15]专题教育，严格落实“八项规定”，大力整治“四风”[16]，市直党政机关和参公单位“三公”经费[17]财政拨款总支出连续五年“零增长”。完成公务用车制度改革。加强基层组织建设，实行普遍直接联系群众制度。成立市决咨委[18]、经济研究中心。“江门信息大道”建设启动。“12345”政府服务热线平台不断完善，共受理有效事项超77万宗，开通“微市长”“邑网通”，开启“邑门式”行政服务[19]改革。强化廉政风险防控，严格落实一岗双责，坚决查处违法违纪案件。

　　同时，我市自身发展还面临不少困难和挑战，突出体现在三块短板上：一是“江门制造”的短板，工业投资偏弱，产业规模不大、集聚度不高，致使经济总量偏小，人均地区生产总值低于全省平均水平；二是“江门创造”的短板，自主创新能力不强，科技含量偏低；三是全面小康的短板，小康指数部分指标与全省对比还有较大差距，基本公共服务水平不高，区域发展不协调矛盾依然突出。此外，行政管理层次多、效率低、权责利不对等问题仍有待破解。我们将采取更有力措施，认真加以解决。

　　各位代表！过去五年政府工作取得的成绩来之不易。在此，我代表市政府，向关心支持江门发展建设的各界人士表示衷心的感谢！

　　“十三五”时期的目标任务

　　“十三五”时期政府工作指导思想是：全面贯彻党的十八大和十八届三中、四中、五中全会精神，以邓小平理论、“三个代表”重要思想和科学发展观为指导，深入贯彻习近平总书记系列重要讲话精神，按照中央“四个全面”[20]战略布局，以及省委“三个定位、两个率先”[21]决策部署，市委“一个同步、五个突破”[22]总体要求，坚持创新、协调、绿色、开放、共享的发展理念，以加快发展为第一位任务，落实“珠西战略”，推动“高、大、中、小”四轮驱动，着力深化供给侧结构性改革[23]，着力推进“双引双创”，着力建设绿色生态美丽家园，着力增进民生福祉，全力打造“三门”、建设“三心”，率先全面建成小康社会，建设干部作为、人民幸福的中国侨都。

　　“十三五”时期我市经济社会发展的主要目标是：经济综合实力实现争先进位，地区生产总值和人均地区生产总值增幅高于全国、全省和珠三角平均水平，城乡居民收入与经济发展同步增长，地区生产总值和城乡居民人均收入比2010年翻一番，人民生活水平和质量普遍提高，全面深化改革取得新突破，区域协同发展呈现新格局，生态文明建设取得明显成效，到2018年与全省同步全面建成小康社会。

　　“十三五”时期是我市创新加快发展和加快创新发展的同步推进期，是城市融合提质的加速期，是动力转换的关键期，更是与全省同步率先全面建成小康社会的决战期。做好“十三五”工作，实现“十三五”目标，任重道远，必须克服中游心态，努力奋勇争先，崇尚创新、注重协调、倡导绿色、厚植开放、推进共享，统筹协调聚合力、双引双创谋动力、深化改革激活力，聚焦聚力聚能做好“五个统筹”。

　　一是在创新发展上统筹创新加快与加快创新。落实“珠西战略”和创新驱动战略，紧盯打造“江门特色、广东标杆、全国示范、国际平台”的全国小微双创之都、全球华侨华人双创之城示范建设，统筹抓好三大平台[珠三角（江门）国家自主创新示范区、广东（江门）大广海湾经济区与中欧（江门）中小企业国际合作区]建设，着力打造五大产业集群[24]（轨道交通、重卡和商用车、新材料新能源及装备、教育装备、大健康等产业集群）。坚持抓大项目，围绕“4+1”产业体系（装备制造、智能制造、绿色制造、精密制造及北斗卫星应用技术），制定江门制造2025规划，重振“江门制造”，推动“江门智造”。加快建设创新驱动发展先行市，力争实现“四个倍增”（R&D占GDP比重、高新技术产品产值占规上工业总产值比重、高新技术企业数和科技型小微企业数、科技孵化器及新型研发机构实现倍增）。建设科教强市、人才强市，实施五邑人才工程，打造人才创新创业高地，制定江门创造2025规划，打造“江门创造”。积极推动“互联网+”，打造“全光网城市”。

　　二是在协调发展上统筹“东联西拓”与“东提西进”。对外继续深化与广佛都市圈、深港澳经济圈两大龙头的主动对接，重点推动“江深合作”“江佛一家”，开拓“江广合作”。加强与珠西各市合作发展，积极参与珠江-西江经济带发展。持续推进大交通建设，加快规划建设“一大枢纽、四大路网”[25]。对内促进资源优化整合，东部着力强中心、拓门户，推动东部一体；西部加快台开同城，培育江门城市的副中心，形成内生动力，带动台开恩在交通、文旅、园区、农业、环保上协同共进。进一步厘清市县镇村权责利关系，市一级重在统筹、协调、监督，特别是统筹好三大平台、五大产业集群的集约集聚发展，县（区）一级重在建设、管理与服务，镇（街）是社会治理的主平台，村（社区）是社会公共服务的末梢与终端，重在社会治理和基层服务，促进上下联动、协调同步。

　　三是在绿色发展上统筹优化主体功能区与强化红线意识。加快形成主体功能区布局，推进“多规合一”，构建既集约高效、又望山见水的空间格局。促进节约集约发展，优化存量，提升质量。持续“种树”，久久为功，建设生态强市，创建国家森林城市，推进公园城市建设，构建市域公园体系。同时，强化红线意识，严格落实生态控制线、林业生态红线、永久基本农田保护红线，严格按照环境容量及总量控制目标进行结构调整和精准招商。加强环境保护和污染治理，巩固国家环保模范城市建设成果，深入实施大气、水、土壤等防治行动，消除劣V类水体，建设秀美侨都。

　　四是在开放发展上统筹整合内外资源与创造良好营商创业环境。把握国家“一带一路”[26]战略实施与中国（广东）自由贸易试验区建设的重大机遇，加强与欧美的对接，探索建立城市联盟。大力建设粤港澳合作新平台，继续推动广东（江门）大广海湾经济区上升为国家级平台。围绕打造市场化、国际化、法治化的营商环境，坚持“市场事，市场定；社会事，社会建；政府事，公平办”，深化和完善现代市场体系、财政体制、投融资体制、行政管理体制等重点领域改革，推动国有经济和民营经济共生互动发展。充分利用好海内外“两个江门”的丰富资源，推动“中国侨都”建设，着力聚侨情、引侨资、借侨智、重侨创，充分调动起加快发展的各方积极性。

　　五是在共享发展上统筹民生普惠与精准托底。持续推进基本公共服务均等化，提高城乡居民收入水平，促进多形式就业，完善社会保障体系，加快发展教育、医疗卫生等社会民生事业，推动公交一体，打造文化强市，建设健康江门，维护社会和谐稳定。进一步抓好精准扶贫、精准脱贫工作，着力提升底线民生保障水平，确保全面小康覆盖全市域的所有人口。建设惠民侨都、秀美侨都、文明侨都、平安侨都，让人民群众共享更多的发展成果。

　　2016年工作安排

　　2016年是“十三五”规划的开局之年，也是挑战巨大的一年、创新加快的一年、项目突破的一年、弥补短板的一年、应变求变的一年。今年政府工作的总体要求是以党的十八大、十八届三中、四中、五中全会、中央经济工作会议、省委十一届五次、六次全会和市委十二届六次全会精神为指导，紧扣“一个中心、两大板块、三大平台、四轮驱动、五大产业”[27]的经济工作主线，坚持创新加快发展与加快创新发展相统一，充分发挥重大平台作用，加快重大工程建设，推动重大项目突破，擦亮小微双创品牌，着力促加快、强中心、补短板、优环境、惠民生，推动江门再出发，为“十三五”开好局、起好步。

　　今年全市经济发展的预期目标为生产总值增长9%，一般公共预算收入增长9%（其他指标见附件）。

　　为实现上述发展目标，重点抓好六大工作任务：

　　一、创新加快，加快创新

　　制造强市。坚持抓大项目，今年是项目推进的突破年，着重围绕三大平台、五大产业集群，集中精力集聚资源，落实敲门招商、中介招商、以商引商，推动精准招商，力求在重大产业项目引进和培育上取得新突破。做好产业规划和链条设计，建立健全从“谋谈签”项目—招商项目库—项目储备库—重点项目库的链式机制，实施对工业投资特别是重大产业项目投资的专项考核，确保今年建成一批、引进一批、新开工一批优质产业项目。对全市产业发展进行统筹整合，蓬江加快建设滨江新城尤其是江沙示范园，统筹打造教育装备产业集群，以大长江、海信等为龙头大力发展先进装备制造、智能制造、生命科学等产业。江门高新区加快发展总部科技园、建设国家创新型特色园区，统筹打造大健康产业集群，做实做强绿色光源、新材料、生物健康等新兴产业，积极培植物联网及北斗应用产业。新会加快大广海湾经济区银洲湖先导区的产业规划发展，统筹打造以广东中车为龙头的广东轨道交通产业集群，并以中集、李锦记（无限极）为龙头加快船舶、海洋装备、精细化工、健康食品等产业集约发展。鹤山以鹤山工业城为主体推动建设鹤新蓬产业合作区，统筹打造中欧（江门）中小企业国际合作区主基地，着力在机械装备、电力设备、专用车、新能源汽车等产业寻求突破。台山加快工业新城建设，以富华重工等统筹打造重卡和商用车、新材料新能源及装备产业集群。开平以翠山湖产业园为重点，打造五金机械、电子信息、新型材料和汽车及零部件产业基地。恩平推动一园三区[28]联动发展，建设广东机械装备产业园。

　　创新争先。抓好珠三角（江门）国家自主创新示范区建设，围绕“珠西智谷”“珠西创城”“冈州创客”等创新载体，强化创业教育，发展创客空间，完善创新发展服务体系，打造以五邑大学为中心的“环五邑大学创新经济圈”，成为全市创新驱动的重大引擎。鼓励大学生众创空间建设，打造广东首个创客街区。大力培育高新技术产业，国家级高新技术企业达240家，推动科技成果实现产业化500项，创建10家市级以上企业工程研究中心，落实保护知识产权措施。加强引进各类研发机构，加快江门中微子实验站进度，组织实施100项市级重点科技计划项目，开展产学研项目80项。研究设立支持实体经济与创新经济发展的政府投资基金，推进科技、金融、产业融合发展。加快科技金融综合服务中心建设，实施专业镇转型升级示范计划。推进小微双创示范工作，全面实施“雏鹰计划”，构筑“创业苗圃—孵化器—加速器—专业园区”孵化链条。推进省小微企业创业创新综合改革试点市建设，大力促进民营经济创新发展，推动“邑商再出发”。进一步完善小微企业和科技型小微企业名录库，推进科技型小微企业认定标准体系建设，探索建立小微企业双创指数。强化金融对企业的支撑，支持金融产品创新服务中心发展，推进多层次资本市场建设，争取多家企业上市，鼓励中小微企业在新三板和股权交易中心挂牌融资，支持企业并购重组。依托全国小微企业信用体系建设试验区，加强金融第三方信用评级工作。出台人才强市政策，开展企业家人才培训，在人才引进、培育智力支撑发展上有新突破，营造“留得住人才、用得好人才”的环境。深入推进“青创汇”、全国博士后创新（江门）示范中心、院士工作站、博士后科研工作站等建设。建设中国（江门）“侨梦苑”华侨华人创新产业聚集区，成立五邑侨智库。结合推动“互联网+”，推进“江门信息大道”“珠西数谷”“珠西云谷”建设，在全市域打造“全光网城市”，推动光纤网络、4G网络、公共场所免费WiFi网络城乡全覆盖，建设江门市电子政务云计算中心和台山市云资源中心。

　　转型升级。围绕“六新六去”[新技术（技改）、新实业、新消费、新金融、新城镇、新农村，去过剩、去库存、去低效、去负担、去污染、去繁就简]，推进供给侧结构性改革。着力淘汰一批过剩、低效、污染的企业、行业，对产能过剩企业采取回购、产业置换、兼并重组等形式提效增能，鼓励低效无效企业进行“棚改厂”“工改工”“单层改多层”，加紧拆改违章建筑厂房，促使污染项目进行环保改造、搬迁或关停，以中心城区功能调整为契机优化企业跨区布局。着力盘活一批存量土地，实现“二次开发”和“二次利用”，建立健全新增建设用地指标分配与盘活存量建设用地挂钩机制，进一步解决和利用好农村集体发展留用地，优先保障制造业项目用地，推进“三旧”改造[29]工作，促进房地产去库存。着力升级一批园区和传统产业，持续推进“七个一”专项行动[30]，提升“1+6”核心园区建设水平；继续落实“六个优先”[31]，推动企业增资扩产，实施“机器人应用”工程，以智能制造、“互联网+”等新技术提升传统产业技术水平，完成技改投资增长25%以上，力促重大技改项目突破，把一批传统企业改造成为高新技术企业，实现“吹糠见米”。加大市属国有资产整合重组力度，积极组建金融控股公司，做精做专国资运营平台。加快外贸转型升级，巩固传统市场，开拓新兴市场，推动跨境电子商务、旅游购物出口等外贸新业态发展，推进江门电子口岸实体建设，提高口岸通关效率。

　　二、做强中心，拓展门户

　　谋划城局。调整完善规划管理体制，充分发挥市规划委员会统领监督作用，落实主体功能区规划，启动全域规划编制，加快重点发展区域的控制性详细规划编制，推进一批城市建设重大项目落地。科学谋划大城市格局，东部三区一市以江门大道为中轴，重点谋划蓬江滨江新城金融商务会展中心、江海科教新城科创中心、新会枢纽新城综合交通枢纽中心、鹤山物流枢纽中心，统筹优化教育、卫生、文化、会展等城市功能布局，进一步明晰城市功能定位，强化中心城区带动力与辐射力，拓展江门南、北“枢纽门户”功能；西部台开恩以台开快速路为突破，着力推动台开恩联城快速路建设，推动县域经济协同发展。强化城乡协作协同，推动专业镇、特色小镇培育发展，推进4个省新型城镇化“2511”试点项目[32]实施，加快省、市级新农村连片6个示范工程建设，创建一批宜业宜创宜居宜游的秀美镇村。不断完善城市管理和服务，加强城市管理数字化平台建设和功能整合。推进户籍制度改革落地，加快提高户籍人口城镇化率。

　　交通先行。大力推进交通大会战，今年是交通建设决战年，江门大道中建设实现五洞-东甲立交段建成通车，规划建设东部联城高快速路网。启动台开快速路建设。确保江罗高速年内通车，加快广中江、中开、高恩高速建设和佛开高速三堡至水口段扩建，开展新会至高栏港、阳春至开平、开平至台山等高速前期工作。做好与港珠澳大桥、深中通道的规划衔接，重点完善中开高速沿线的三江、双水、水步、百合等关键节点，

　　全力打造“深（中）江通道”，提升江门作为广佛、深港澳辐射粤西及大西南的门户战略地位，加快形成东西板块互联互通的大江门发展格局。完成珠西综合交通枢纽江门站立项，调整站场规划布局，推进深茂铁路江门段、南沙港铁路江门段建设，加快深茂铁路江门至深圳段、广佛江珠城轨等项目前期工作，积极谋划江肇高铁，编制城市轨道交通线网规划。加快推进高新区码头建设、广海湾港区码头与“空港”规划。推进公交一体，完善东部直达快线、快速公交、常规公交、微循环公交4个层次公交系统建设，打造智慧公交，实施车厢内部、候车亭无线上网。加快公交枢纽站综合开发建设，推进公共自行车项目、停车场的规划建设。推广新能源汽车在公共服务领域应用。

　　融合提升。重点推动“江深合作”，搭建“龙头+基地”模式，主动对接深圳科技、先进制造业、生产性服务业，促进深度合作。推进江佛融合和珠中江阳[33]区域紧密合作，谋划“江广合作”模式新突破，着力加强在交通、产业、文旅上的主动对接。推动建立全国小微企业创业创新基地示范城市联盟，探索建立中欧（美）小微双创城市联盟。推进江港澳更紧密合作，加快推进粤港澳服务贸易自由化省级示范基地建设，落实江澳金融合作备忘录。

　　三、强农固基，文旅兴城

　　农业强市。打造开平市国家现代农业示范区、粤台（江门）农业合作试验区和广东农产品加工示范区等现代农业发展平台，加大农业龙头企业等新型农业经营主体的扶持培育力度，力争新增3家农企挂牌上市，办好第五届农业博览会。培育新型职业农民，推动农业产业化。启动农产品质量安全示范市创建，加快建设农产品溯源体系。推进新会国家农村综合改革示范试点和农村土地承包经营权确权登记颁证工作，建设农村产权流转管理服务平台，加强农村集体“三资”[34]监管。开展第三次全国农业普查工作。抓好永久基本农田划定工作。落实粮食安全责任制。全面完成25.5万亩和启动新建22.4万亩高标准基本农田建设任务。创新资金投入方式，引进社会资本，加快水利设施和“三防”基础建设。加快渔船更新改造，组建远海捕捞船队，推进台山烽火角避风塘和广海渔港建设。建设“美丽乡村”，新增开展63条行政村村庄整治。

　　旅游强市。争创全国全域旅游示范市，进一步做好台开恩世遗文化旅游度假区创建中国国际特色旅游目的地申报工作，打造中国休闲旅游之城。启动环圭峰山国家级旅游集聚（实验）区创建，围绕白沙文化打造“岭南心学第一山”。抓好景区创A工程，力争开平碉楼文化旅游区成功创建国家5A级景区。做好旅游龙头重点项目招商，重点抓好开平赤坎古镇、台山滨海旅游、恩平航空小镇、蓬江文化创意产业园、新会美丽湖湾等龙头项目的招商建设。构建江门城市客厅[35]，按照“主客共享”全域旅游理念，抓好旅游驿站、温泉旅居、旅游特色街区、特色民宿、步行径等建设工作。推动“醉美江门100村”乡村旅游建设，开展乡村旅游示范镇村工程。

　　文化强市。制定文化强市行动计划，擦亮“世遗文化?中国侨都”城市文化品牌，加强邑商文化研究，办好第五届世界江门青年大会，建设广东省华侨文化经济合作创新示范市。完善江门水道沿线规划，调整水系调度管理体制，加快蓬江“33墟街”、江海“南岸印迹”、新会“学宫文心”协同规划建设，谋划鹤山“岭南鹤武”，推动文化振兴、以文融城。开展历史文化名村和传统村落申报，推动自然村落历史人文普查工作。推进市县镇村四级文化教育场馆整合，完成文化馆、美术馆建设，推进华博馆、图书馆升级改造，构建江门五邑联合图书馆、文化馆、博物馆、美术馆，带动全市各级各类文化资源的融合提升。推动市档案馆新馆筹建和各区（市）档案馆全面达标工作。征集出版《五邑侨胞耀中华》史料。加快现代公共文化服务体系建设，全市镇（街）综合文化站 100%达到省一级站标准。继续实施文化下乡、文化惠民工程。推进海上丝绸之路史迹点申遗。完成第一次全国可移动文物普查。加快工艺美术、印刷发行等文化体育产业发展，推进版权兴业工作。

　　四、绿色发展，秀美侨都

　　公园城市。抓好创建国家森林城市工作。初步完成圭峰山、白水带、龙舟山、大雁山、石花山、梁金山、鳌峰山等龙头公园的整合和建设，加快台山中国农业公园建设，规划建设蓬江天沙湖、江海龙溪湖、新会思成湖及万亩小鸟天堂、鹤山古劳水乡、台山大隆洞、开平大沙河、恩平沙湖等湿地公园，打造各区（市）至少有“一山一水”的公园格局。新建改建443个村居公园，新增17个森林公园，建成21个镇级公园、6个综合性公园，加快江门市植物园和7个特色田园风光公园规划建设，加强文化主题公园建设，新建慢行系统和城乡绿廊90公里。完成各级公园建设标准编制和公园统一标识。继续坚持“种树”，积极推进饮用水源水库第一重山商品林调整为生态公益林工作，完成调整5.17万亩。推进乡村绿化美化工程建设。深化集体林权制度改革。加快森林防火基础设施建设。

　　治理环境。推进多污染物综合防治，加大水环境综合整治力度，加快杜阮污水处理厂管网二期工程（续建）项目进度，进一步推进建制镇和全面启动农村污水处理设施规划建设，全市城镇污水处理率达到91%。进一步改善大气环境质量，加强机动车污染防治，全面实施汽油车国五排放标准。加强重金属、土壤、固体废物污染防治力度。落实最严格的水资源管理制度，强化饮用水源保护，推进潭江和天沙河、麻园河、会城河等重点河涌综合整治，大力整治污染源。完成市区应急备用水源及供水设施工程建设。完善城乡生活垃圾收运体系，扩大生活垃圾收运范围，促进垃圾减量处理，全市农村生活垃圾有效处理率达90%以上。加强环境监管执法，建立全市重点监控企业“一企一档”动态数据库。

　　低碳发展。推进节能减排降耗，淘汰落后产能，促进工业园区循环化改造，加强产业园区热电联产项目建设。积极开展排污权交易试点，实施主要污染物排放总量控制。落实最严格的节约用地制度和最严格的耕地保护制度。建设“海绵城市”[36]，推进海绵型道路和广场建设。加强地下综合管廊建设。

　　五、民生为本，共建共享

　　加大民生投入，全市安排民生支出207亿元，同比增长23%。进一步巩固创文成果，深化“大民政”工作，深入推进基本公共服务均等化综合改革，抓好十件民生实事落实（见附件），让广大市民群众得到更多实惠。

　　服务均等。完善城乡社区服务体系，推进村（社区）公共服务站建设，做实做好“邑家园”家庭综合服务中心，努力实现“邑家园”综合服务平台全覆盖。推进光纤入社区、农村（农户）和农村应急广播、WiFi全覆盖到行政村工作，抓好礼乐向民村试点建设。引导社会组织规范有序发展，重点培育、优先发展行业协会商会类、科技类、公益慈善类、城乡社区服务类社会组织，引导社会组织参与社会治理。

　　精准扶贫。统筹城乡精准扶贫工作，建立常态化全覆盖扶贫长效机制，将全市有劳动能力的城乡低保家庭纳入精准扶贫、精准脱贫帮扶范围。加快城乡贫困对象建档立卡，推进扶贫对象全公开，建立“一户一策”、对口帮扶的“就近扶贫”模式，开展百企扶百村、百医牵百村、千（万）义工助千户行动，支持慈善机构参与扶贫开发，努力实现精准脱贫。继续实行基本公共服务均等化补助和资源激励型财政补贴政策，村委会集体收入进一步提高至15万元以上。加快综合社会救助体系建设，提高全市人均城乡低保补差水平、特困人员供养标准以及孤儿和艾滋病病毒感染儿童供养水平。医疗救助对象政策范围内住院自付费用医疗救助比例提高到80%以上。做好适度普惠型儿童福利制度试点工作。加快推进残疾人小康进程，扶持300户困难残疾人家庭发展生产和自主创业。完成棚户区和农村危房改造任务。

　　就业保障。实施精准化就业帮扶，鼓励多渠道多形式就业，全市城镇新增就业4.5万人以上，城镇失业人员再就业3万人，应届高校毕业生就业率达90%以上。完善就业创业政策，推进创业孵化基地建设，树立创业导师品牌。推进“五险一金”[37]精简归并，进一步提高社会保险各项待遇，拓展社会保障卡功能应用，加强社保基金监管，不断完善医保付费方案。稳步推进机关事业单位养老保险制度改革。扩大完全被征土地农民基本养老保障工作试点范围。

　　教育提质。科学完善学校布局，优化教育资源配置，加强智慧云信息化建设。实施学前教育扩容提质工程，积极推进学前教育公益普惠优质发展。大力促进义务教育优质均衡标准化发展，推动普通高中教育优质特色多样化发展。增强高等教育服务地方经济社会发展能力，支持五邑大学建设国内知名、全省一流的工科院校，开门办学，推动校企合作、校地合作、校校合作，规划建设轨道交通学院，江门职业技术学院通过省示范校验收，加快推进广东省江门市幼儿师范高等专科学校和广东省江门市中医药职业学院建设。完成市启智学校扩建工程，推进国家级特殊教育改革实验区项目实施。深化产教融合，推进现代职业教育结构优化，加快构建现代职业教育体系。加大对民办教育扶持激励力度，加快民办教育规范特色优质发展。构建完善的终身教育服务体系。加快高素质专业化教师队伍建设，大力推进教育名师工程。

　　健康江门。制定卫生强市、卫生强县行动方案，重点实施“强基创优”行动计划，完善医保、医疗、医药“三医”联动机制，打造“健康江门”。深化公立医院体制改革，推进分级诊疗体系建设。进一步加强基层医疗卫生服务体系建设，推进乡镇卫生院及村卫生站建设。大力推进社会办医，完善落实社会办医政策措施。加快全市区域卫生信息平台建设。抓好卫生城市、卫生镇复审和创建工作，大力推进农村户厕改造。推进从计划生育、生育管理到生育计划、生育服务转变，落实“全面二孩”政策。加快推进江门体育中心、广东银葵医院等项目建设。发展养老健康产业，完善养老服务体系建设。进一步完善食品药品监督体系建设，推动市食品、药品检验检测机构挂牌运行。加强重大疾病防控，完善疾病预防控制机构三级网络。建设江门市国民体质检测中心，加强社区体育设施建设，办好第八届市运会。

　　力保平安。坚持社会治安综合治理，加快创新社会治安防控体系，严厉打击群众反映强烈的突出犯罪。深入推进警务大数据平台建设，搭建警务云一期基础云服务平台，建设完善可视化指挥调度平台。深入推进社区警务，大力深化“平安社区”建设。建设完成二级互联网信息监控中心和“网安警务室”。树立安全发展理念，强化城市安全意识。实行维稳及安全生产党政同责、一岗双责、失职追责。抓好重点领域事故隐患排查整治，坚决遏制较大以上事故发生。完善突发事件预警信息发布体系。加强应急管理建设，推进应急救援队伍能力提升，进一步普及全民应急意识。

　　此外，进一步完善国防动员体系，落实双拥和优抚安置工作。继续做好民族、宗教、对台、人防、打私、统计、方志、妇女儿童、红十字会等工作。

　　六、法治政府，透明提效

　　依法行政。自觉维护宪法与法律权威，严格依照法定权限和程序行使权力、履行职责，建设法治政府。积极配合人大做好地方立法工作，全面启动政府规章的制定工作。主动接受人大监督，认真执行市人大及其常委会各项决议决定，支持政协委员参政议政，提高人大代表议案、建议和政协提案办理水平。完善重大行政决策法治化程序。探索开展实施中期财政规划和三年滚动预算试点，加强政府债务限额管理，防范系统性风险。加强政府法律顾问工作，完善政府法律顾问制度和一村（社区）一法律顾问制度，加强律师公益法律服务工作。全面启动第二批司法体制改革试点工作。严格规范公正文明执法。启动“七五”普法规划。积极开展法治创建活动，全市50%以上镇（街）创建法治镇（街），提升基层社会治理法治化水平。

　　公平透明。加强政务公开工作，继续完善全口径财政预决算、预算调整、部门预决算以及“三公”经费预决算信息公开，探索行政执法公开、第三方评估政府行政许可绩效。深化公共资源交易体制改革，着力构建“全市一张交易网”，推进交易全过程电子化，逐步实现公共资源交易全覆盖。建立健全普遍直接联系群众制度。着力加强政务网站、微博群和微信公众号建设，完善政府新闻发布制度，开展网络问政及微访谈。强化公有资产在经济社会发展中的先导作用，鼓励在公共服务领域推广政府和社会资本合作模式，积极发展混合型经济，推进PPP合作模式[38]项目。完善政府投资基本建设项目管理体制，加强投资规划调控。打造“信用江门”，建立健全社会信用体系，推进政务诚信、商务诚信、社会诚信和司法公信建设。

　　行政提效。进一步降低门槛、降低企业负担，提升政府服务水平，推动“暖企行动”常态化，实行“收费就低不就高、办事就简不就繁、服务就近不就远”。全面深化商事制度改革，推进“多证合一、一照一码”“全程电子化登记”，强化事中事后监管。进一步落实“1+3”清单应用实施，出台第二批负面清单，建立健全行政审批中介服务收费目录清单管理制度，开展行政审批标准化建设工作。构建政务信息资源目录体系，建设统一信息资源库和管理平台，推行首席数据官、数据专员制度，组建网络信息安全监测和安全中心,加快政府数据开放共享。提升“12345”政府服务热线水平，推广和深化“邑门式”服务，打造完善江门市政务大数据管理平台、统一网上行政审批服务平台、统一应急指挥中心平台、“邑网通”、“微市长”、“侨都之窗”等政务大数据应用示范工程，宁可数据在网上多跑几圈，也要让企业和群众少跑一轮。

　　奋发有为。抓好政府系统党风廉政建设，严肃查处顶风违法违纪案件，落实一岗双责，坚持厉行节约，严控“三公”经费支出。完善监察、司法、审计等联动的行政权力监督机制。加大督促检查力度，健全绩效考核和监督问责制度，严肃整治行政“不作为、乱作为、慢作为”现象，增强大局意识、实干意识、服务意识，形成想干事、会干事、干成事的氛围。强化政府创新能力、协同能力、应变能力、化险能力、法治能力建设，着力打造一支充满激情、勇于担当、干净干事的公务员队伍。

　　各位代表，同志们，我市“十三五”发展蓝图已经绘就，落实的关键在人、在作风、在执行。让我们团结一致、奋力拼搏，不辜负省委、省政府和江门人民的新要求和新期望，打造“三门”、建设“三心”，建设幸福中国侨都！
